# *Parachlamydia acanthamoebae* Detected during a Pneumonia Outbreak in Southeastern Finland, in 2017–2018

**DOI:** 10.3390/microorganisms7050141

**Published:** 2019-05-17

**Authors:** Kati Hokynar, Satu Kurkela, Tea Nieminen, Harri Saxen, Eero J. Vesterinen, Laura Mannonen, Risto Pietikäinen, Mirja Puolakkainen

**Affiliations:** 1 Department of Virology, University of Helsinki and Helsinki University Hospital, FI-00014 Helsinki, Finland; Satu.Kurkela@helsinki.fi (S.K.); Laura.Mannonen@hus.fi (L.M.); Mirja.Puolakkainen@helsinki.fi (M.P.); 2 Children’s Hospital, University of Helsinki, FI-00029 Helsinki, Finland; tea.nieminen@hus.fi (T.N.); harri.saxen@helsinki.fi (H.S.); 3 Department of Ecology, Swedish University of Agricultural Sciences, SE-75007 Uppsala, Sweden; ejvest@utu.fi; 4 Biodiversity Unit, University of Turku, FI-20014 Turku, Finland; 5 Department of Internal Medicine, Kymenlaakso Central Hospital, FI-48210 Kotka, Finland; Risto.Pietikainen@kymsote.fi

**Keywords:** *Chlamydia-related bacteria*, *Parachlamydia acanthamoebae*, *Chlamydia pneumoniae*, pneumonia, respiratory tract infections, disease outbreak, nucleic acid amplification test

## Abstract

Community-acquired pneumonia (CAP) is a common disease responsible for significant morbidity and mortality. However, the definite etiology of CAP often remains unresolved, suggesting that unknown agents of pneumonia remain to be identified. The recently discovered members of the order Chlamydiales, Chlamydia-related bacteria (CRB), are considered as possible emerging agents of CAP. *Parachlamydia acanthamoebae* is the most studied candidate. It survives and replicates inside free-living amoeba, which it might potentially use as a vehicle to infect animals and humans. A *Mycoplasma pneumoniae* outbreak was observed in Kymenlaakso region in Southeastern Finland during August 2017–January 2018. We determined the occurrence of Chlamydiales bacteria and their natural host, free-living amoeba in respiratory specimens collected during this outbreak with molecular methods. Altogether, 22/278 (7.9%) of the samples contained Chlamydiales DNA. By sequence analysis, majority of the CRBs detected were members of the Parachlamydiaceae family. Amoebal DNA was not detected within the sample material. Our study further proposes that Parachlamydiaceae could be a potential agent causing atypical CAP in children and adolescents.

## 1. Introduction

Community-acquired pneumonia (CAP) is a common disease responsible for significant morbidity and mortality. A variety of micro-organisms, including bacteria and viruses, can cause CAP. The most common bacterial pathogen in typical (sudden-onset) CAP is *Streptococcus pneumoniae*. The more slowly developing atypical CAP presenting often with extrapulmonary symptoms can be caused by *Mycoplasma pneumoniae*, *Chlamydia pneumoniae*, *Legionella pneumophila*, or viruses. Despite recent advances in microbiological techniques, the definite etiology of CAP often remains unresolved.

*Chlamydia pneumoniae* belongs to the Chlamydiaceae family within the order Chlamydiales, a group of obligate intracellular bacteria featuring a biphasic developmental cycle. Chlamydiaceae contains also well-known veterinary pathogens *Chlamydia psittaci* and *Chlamydia pecorum* that are known to be zoonotically transmitted to humans from infected animals (birds and livestock, respectively) and can cause pneumonia. In addition to Chlamydiaceae, the order Chlamydiales contains a wide and evolving set of novel bacteria, recently suggested to be taxonomically classified in 13 additional families within the order and collectively called Chlamydia-related bacteria (CRB) [1]. Many of the Chlamydia-related bacteria naturally reside in free-living amoeba, and CRB have been detected in a wide variety of environmental samples such as water and soil [2], but also in a wide range of animals, insects and arthropods [3]. Several of the CRBs have been suggested to cause respiratory disease and pneumonia in humans. The role of *Parachlamydia acanthamoebae* as an agent of pneumonia is most studied and many of the modified Koch’s postulates are fulfilled. For instance, the pathogenicity is suggested by sero-epidemiological and molecular studies as well as by an experimental pneumonia model including a treatment trial [4,5]. *Waddlia chondrophila* is mainly associated with tubal factor infertility [6] and adverse pregnancy outcome [7], but also with lower respiratory tract infections [4,8,9]. In some reports, *Simkania negevensis* and *Rhabdochlamydia* spp. are detected in association with respiratory infections [10,11,12]. In our previous study, *Rhabdochlamydia* spp. DNA was detected in 12% of respiratory specimens from Finnish patients with respiratory tract infection [11].

A *M. pneumoniae* outbreak was observed in Kymenlaakso region in Southeastern Finland during August 2017–January 2018 [13]. The aim of this study was to study occurrence of Chlamydiales bacteria in respiratory specimens collected during this outbreak with molecular methods targeting the Chlamydia-related bacteria and their natural host, free-living amoeba.

## 2. Materials and Methods

### 2.1. Respiratory Tract Samples

The respiratory swab samples analysed in this study were collected during a *M. pneumoniae* outbreak observed in Kymenlaakso region in Southeastern Finland during August 2017–January 2018 (as described in detail in [13]). Respiratory tract swab specimens were available from two groups, from a total of 278 individuals. The first group contained 96 individuals who sought publicly funded healthcare consultation at local GPs or hospitals in the Kymenlaakso region and who were subsequently tested for *M. pneumoniae* nucleic acid between 1 August 2017 and 31 January 2018. The second group contained 182 individuals who participated in school screening arranged in four schools in the same area (Virolahti Middle School, Virojoki School, Virolahti High Shool and Klamila School, in Kymenlaakso region, Southeastern Finland). To collect information on their symptoms, individuals in the second group were asked to fill in a questionnaire. According to the survey, 112 had respiratory symptoms and/or fever, 15 did not report such symptoms, and from 55 individuals the information was not available. 

### 2.2. DNA Extraction

DNA was extracted from respiratory specimens using MagNA Pure LC Total Nucleic Acid Isolation Kit (Roche Diagnostics GmbH, Mannheim, Germany). In detail, 300 μL of specimen was lysed with 300 μL of MagNA Pure Lysis/Binding Buffer (Roche Diagnostics GmbH, Mannheim, Germany). Five hundred μL (MagNA Pure LC) of specimen lysate was extracted with the MagNA Pure LC and eluted in 50 μL of the elution buffer.

### 2.3. Detection of Chlamydia pneumoniae

A multiplex real-time PCR assay for simultaneous detection of *M. pneumoniae*, *Chlamydia pneumoniae* and mutations associated with macrolide resistance in *Mycoplasma pneumoniae* 23S rRNA gene along with an internal control developed by us [14] was used (Table 1). This assay amplifies *C. pneumoniae* ompA gene [15], and the analytical sensitivity (limit of detection with 95% probability) was 0.13 IFU/PCR reaction [14].

### 2.4. PanChlamydiales-PCR

Specimens were screened with a Chlamydiales-specific amplification method (PanChlamydiales-PCR) as described earlier by Lienard et al. 2011 [16], with slight modifications [17] (Table 1.). This PCR method amplifies a ∼200-bp fragment of the 16S rRNA gene and has been shown to detect a wide range (at least all 15 chlamydial reference strains tested) of different members of the *Chlamydiales* order. A PCR mixture of 25 μL contained 12.5 μL Maxima Probe/ROX qPCR master mix (2X) (Thermo Fisher Scientific, Waltham, MA, USA), 100 nM primers panCh-Fwd and panCh-Rev, and 100 nM panCh-Probe (Table 1). The primers and probe were purchased from Integrated DNA Technologies. Amplification was performed with a 7500 Real Time PCR system (Applied Biosystems, Foster City, CA, USA Negative (water as extraction control and PCR reaction without template) and positive (DNA extracted from *Simkania negevensis* VR-1471) controls were included in each amplification run.

Extreme precautions were taken to avoid contamination in PCR. DNA extraction and preparation of PCR reaction mixes were conducted in separate laboratories with separate equipment, dedicated to pre-PCR activities only. In each run, 2 wells of PCR mix only were included as negative controls and 8 wells were used as nucleic acid extraction negative controls. Cycling conditions were 10 min at 95 °C, followed by 45 cycles of 15 s at 95 °C and 60 s at 60 °C, all carried out in an Applied Biosystems^®^ 7500 Real-Time PCR System (Applied Biosystems, Foster City, CA, USA). All samples were tested in duplicate. Amplicons were sequenced. Samples with Ct ≤ 37 or with Ct 37–40 and a Chlamydiales family levels sequence (> 90% similarity with established Chlamydiales sequence [18]) were considered positive.

### 2.5. Amoeba PCR

All samples giving a signal in PanChlamydiales-PCR were studied further by PCR methods targeting free-living amoeba, the natural hosts of CRB. The primers and probes used for amoeba detection are presented in Table 1 [19,20,21]. The PCR reactions (total volume 25 µL) contained 12.5 µL Maxima Probe/ROX qPCR Master Mix (2X) (Thermo Scientific, Waltham, MA, USA), and 5 µL template DNA. For Acanthamoeba and Vahlkampfiidae (including Naegleria spp.), a duplex PCR reaction, 500 nM of primers and 250 nM probe were used. For Vermamoeba PCR reaction, 200 nM of both primers and probe were used. PCR was performed with 7500 Real-Time PCR system (Applied Biosystems). The cycling conditions were 95 °C for 10 min and 45 cycles of 95 °C for 15 s and 60 °C for 1 min.

### 2.6. Sequencing and Phylogeny

The pan-*Chlamydiales* qPCR amplicons were purified by using an Illustra ExoProStar 1-Step kit (GE Healthcare, Buckinghamshire, United Kingdom) according to the manufacturer’s instructions. Both the forward and reverse strands of the PCR products were sequenced by using primers panFseq and panRseq [16] in the sequencing unit of the Institute for Molecular Medicine Finland. Sequences were compared to the known sequences in the NCBI database by BLAST analysis. For family level classification, the first established CRB strain of the BLAST hit list was determined, and if the sequence identity was ≥90%, the two sequences were considered as members of the same CRB family [17,18,22]. The *Verrucomicrobium spinosum* sequence was included as an outgroup (GenBank accession number X90515), as it was used previously as an outgroup for all *Chlamydiales* [23]. We constructed a Bayesian posterior-probability consensus tree using a total chain length of 1,100,000 and a burn-in length of 11,000. The analysis was carried out by using Geneious software [24]. Sequences were aligned by using the MUSCLE plug-in [25], and Bayesian analysis was done with the MrBayes plug-in [26].

## 3. Results

### 3.1. Detection of Chlamydia pneumoniae

All samples were studied by a PCR based method amplifying *Mycoplasma pneumoniae* and *Chlamydia pneumoniae* [14]. Of the 278 samples, only one (1/278, 0.4%) was shown to contain *Chlamydia pneumoniae* DNA. This sample was from a patient in group 1 (who sought health care consultation with signs and symptoms suggestive of *M. pneumoniae* infection) (sample ID 1410, Table 2).

### 3.2. Detection of Chlamydiales DNA

Altogether 22/278 (7.9%) samples gave a positive result by PanChlamydiales PCR. Of these 22 samples, 5 were from symptomatic individuals that belonged to the group 1 (sought health care consultation). Of the 17 Chlamydiales DNA-positive samples in group 2 (school screening), 11 were from individuals with respiratory symptoms (rhinitis and/or cough and/or fever) and one from those without such symptoms (9.8% and 6.7%, respectively). However, the interpretation is limited by the small number of those reporting no symptoms (*n* = 15). No information on possible respiratory symptoms or fever was available from five individuals with Chlamydiales DNA.

As expected, the specimen positive for *C. pneumoniae* DNA (ID 1410, group 1) was amplified also by PanChlamydiales primers and was included in the set of 22 positive specimens. Of these 22 samples, only two contained also *M. pneumoniae* DNA (sample no V8, group 2 and 1563, group 1). In addition, one individual had serological evidence of a recent *M. pneumoniae* infection (ID 1463, group 1) (Table 2).

### 3.3. Amoeba PCR

Amoebal DNA (Acanthamoeba, Vahlkampfiidae, including Naegleria, or Vermamoeba) was not detected in any of the PanChlamydiales-positive samples (0/22).

### 3.4. Sequencing and Phylogeny

By sequence analysis, 16 (72.7%) of the 22 samples containing Chlamydiales DNA were classified as members of the family Parachlamydiaceae. By this method, sequences belonging to Criblamydiaceae family were identified in two (9.1%) and to Simkaniaceae family in one (4.5%) of the 22 respiratory samples. One sample contained a sequence (4.4%) that was classified as *C. pneumoniae*: by BLAST analysis, the DNA sequence of the PanChlamydiales-PCR amplicon showed 99% identity with the *Chlamydia pneumoniae* genome assembly YK41, GenBank no LN849050. The same sample was identified as positive by the *Chlamydia pneumoniae*-specific PCR. In two (9.1%) samples, the DNA sequences were shown to belong to Chlamydiales order, but remained unclassified on family level.

**Figure 1:**
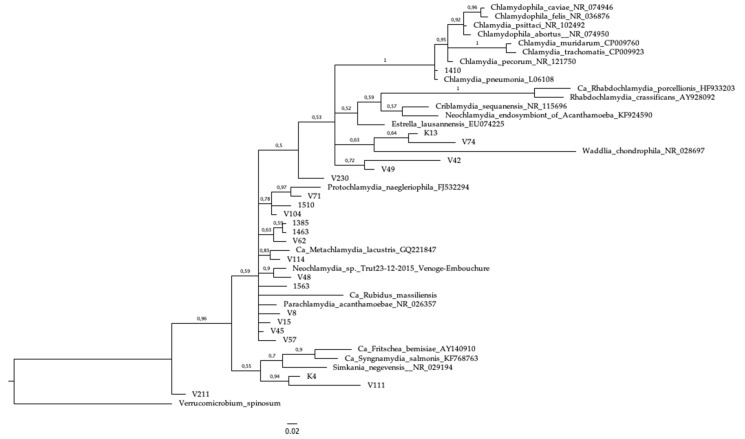
Bayesian posterior probability tree of the sequences in this study along with reference sequences and *Verrucomicrobium spinosum* as an outgroup to root the tree. Some clades are well resolved whereas some are weakly, probably due to short sequence length. The scale bar represents 2% genetic divergence.

In the phylogenetic tree, the *C. pneumoniae* sequence grouped together with the reference sequences from the Chlamydiaceae family. Interestingly, the two unclassified Chlamydiales sequences (V49 and V42) grouped together in the phylogenetic tree, and formed a cluster separate from the Chlamydiaceae family (Figure 1). This might indicate a previously undescribed Chlamydiales bacteria, although the support for this clade is rather low (72%). The two individuals were both from the same school (Virolahti Middle School) and same class (a 14-year old male and a 14-year old female). The boy was reported to have fever and the girl had cough and rhinitis. Some other sequences, such as K4 and V111 may also be examples of novel species, with high support for the clade (94%). The *C. pneumoniae* specific-PCR did not amplify these specimens, although the internal control was amplified.

## 4. Discussion

Starting in 2017, an increasing incidence of respiratory infections, including pneumonias, were observed among school-children and their family members in Kymenlaakso region in Southeastern Finland. Testing of respiratory swab and serum samples from 327 individuals by a PCR method [14] and serology, respectively, provided evidence of *Mycoplasma pneumoniae* infection in 19% of the school-children and their family members [13]. To further investigate the cluster of respiratory infections, respiratory tract swabs from 96 of these individuals (mainly from children) and additional 182 swabs collected from children attending schools in the area were amplified by Chlamydiales-PCR. 

With the PanChlamydiales-PCR assay [16], we detected Chlamydiales DNA in 22 respiratory samples. By subsequent sequence analysis, only one was shown to belong to the Chlamydiaceae family, and it was further classified as *C. pneumoniae*, an established cause of respiratory infections, including atypical pneumonia. The sequencing result was consistent with the *C. pneumoniae*-positive PCR data strengthening the plausibility of the results obtained with PanChlamydiales-PCR and sequencing. Among specimens collected during this outbreak, neither *Chlamydia psittaci* nor *Chlamydia caviae*, which recently have been reported to cause pneumonia in humans, i.e., due to exposure to ill guinea pigs, were seen [27,28,29]. Interestingly, however, two of the sequences that, by the scheme used, remained as “unclassified”, bunched together in the phylogenetic analysis (sequences V42 and V49; Figure 1) and might represent a novel type of Chlamydia-related bacteria. Both samples were from symptomatic 14-year-old individuals, class mates (male and female) from the same school. 

*Parachlamydia acanthamoebae* is the most studied member of the chlamydia-related bacteria, including several studies presenting evidence supporting its role as a CAP causing pathogen [4,5,30,31]. In vitro, Parachlamydia is indeed able to enter and grow in cells present in respiratory tract, macrophages [32] and pneumocytes [33]. Majority of the chlamydiales sequences within our respiratory tract specimens (73%) were taxonomically classified as members of the Parachlamydiaceae family, which is in concordance with earlier studies. Casson et al. set up a real-time PCR method specific for *Parachlamydia acanthamoebae*, and as they applied it to 39 nasopharyngeal aspirates obtained from children with respiratory syncytial virus-negative bronchiolitis, they found 13/39 to contain *P. acanthamoebae* DNA [34]. However, only one of their results could be confirmed by sequencing. Lienard et al. used the PanChlamydiales PCR and subsequent sequence analysis and detected Parachlamydiaceae DNA in 26/422 (6.2%) nasopharyngeal swab samples taken from hospitalized children (between 1–15 years old) [16]. This is in good agreement with our data, as in our study the prevalence of parachlamydiaceae DNA was 5.8 % among respiratory specimens collected from patients between 6–17 years old. In our study 63% of the samples containing Parachlamydiaceae DNA were from individuals with respiratory symptoms and/or fever. In the study by Lienard et al., Parachlamydiaceae DNA was more often detected among patients without (60%) than with (40%) pneumonia [16]. However, as their group without pneumonia were hospitalized children, they supposedly were not completely asymptomatic. In contrast to these data, among 564 adult patients (median age of 76 years; range 21–101 years) with moderately severe community-acquired pneumonia, oropharyngeal samples were studied again by the PanChlamydiales PCR method, and only one was found to contain Chlamydiales DNA [35,36]. Similarly, in our earlier study on respiratory samples from patients sent for *C. pneumoniae* and *M. pneumoniae* nucleic acid testing, *P. acanthamoebae* DNA was not detected (*n* = 102) [11]. However, *Rhabdochlamydia* spp. DNA was detected in 16/136 (12%) of samples studied. The median age of these *Rhabdochlamydia* spp. positive patients was 52 (range 11–72). Thus, the results may reflect a difference among the study populations (adults vs. adolescents), or endemic/epidemic outbreak(s) of different members of Chlamydiales order.

There are no general treatment recommendations for conditions associated with *P. acanthamoeabae*. The antimicrobial susceptibility of Parachlamydiaceae, tested in amoebae, was similar to that of other Chlamydiales, and treatment with macrolides was recommended for parachlamydial pneumonia [37]. As macrolides are also included in the treatment options of pneumonia due to *M. pneumoniae*, most likely also the individuals with *P. acanthamoebae* and other Chlamydiales bacteria benefitted from such therapy.

Even today in as many as 50% of the cases the microbe causing pneumonia remains unidentified. For example, as pointed out by Polkinghorne et al. [27], there is an increasing number of reports on CAP cases caused by bacteria belonging in the genus Chlamydia that thus far have been considered solely animal pathogens, i.e., *Chlamydia caviae*. In addition to members of chlamydiaceae, a wide range of the newly detected chlamydia-related bacteria have been associated with respiratory symptoms including pneumonia. This study further confirms that Parachlamydiaceae can be included in the list of potential agents causing atypical CAP in children and adolescents.

## Figures and Tables

**Table 1:** Primers and probes used in this study.

Target	Primer/Probe	Sequence	Reference
*M. pneumoniae* Hypothetical protein C985_0367	MP Fw	ATGTACTATCAGCAAAAGCTCAGTATGG	[14]
	MP Rev	CCACATACCGCTTTAAGTTAGCAA	
	MP probe	Cy5-CTAACCAAAACAGCCCTTCAACGGCA-Iowa black RQ-Sp	
*C. pneumoniae* ompA	CP Fw	AAGGGCTATAAAGGCGTTGCT	[14,15]
	CP Rev	TGGTCGCAGACTTTGTTCCA	
	CP probe	Tx Red-TCCCCTTGCCAACAGACGCTGG-Iowa black RQ-Sp	
Homo sapiens beta-globin chain	BG Fw	GGTTGGGATAAGGCTGGATTATT	[14]
	BG Rev	CAGGAGCTGTGGGAGGAAGA	
	BG Probe	JOE/ZEN-CAAGCTAGGCCCTTTTGCTAATCATGTTCA-Iowa black FQ	
*M. pneumoniae* gene for 23S rRNA	MR–MP Fw	GCTATAGACTCGGTGAAATCCAGG	[14]
	MR–MP Rev	GCTACAGTAAAGCTTCACTGGGTC	
	MR–MP SimpleProbe^®^	GCGCA XI ACGGGACGGAAAGAC	
Chlamydiales 16S rRNA gene	panCh-Fwd	CCGCCAACACTGGGACT	* [16,17]
	panCh-Rev	GGAGTTAGCCGGTGCTTCTTTAC	
	panCh-Probe	FAM-CTACGGGAGGCTGCAGTCGAGAATC-BHQ1	
*Acanthamoeba* spp. 18S rRNA gene	Acan Fw	CCAGATCGTTTACCGTGAA	* [19]
	Acan Rv	TATTAATGCCCCCAACTATCC	
	Acan probe	FAM-TCTGCCACCGAATACATTAGCATGG-Iowa black FQ	
Vahlkampfiidae spp. 18S rRNA gene	Vahl Fw	GTATAGTCGCAAGACCGAAAC	* [20]
	Vahl Rv	CAAGACAGATCACTCCACGA	
	Vahl probe	Cy5-GGGAAACTCACCAGGTCAGGACAC-Iowa black FQ	
*Vermamoeba vermiformis* 18S rRNA gene	Verm Fw	TACGAGGTCAGGACACTGTG	* [21]
	Verm Rv	ACCATCCGGAGTTCTCG	
	Verm probe	JOE-AATTCCTAGTAGGCGCGAGTCATCAA-Iowa black FQ	

* Modified after.

**Table 2:** Samples containing Chlamydiales DNA.

Sample ID	Age (Years)	Gender	GROUP *	School **	Symptoms		Chlamydiales Family	Other
1410	8	male	1		yes		Chlamydiaceae	*C. pneumoniae* PCR+
1563	10	female	1		yes		Parachlamydiaceae	*M. pneumoniae* PCR+, IgM+
1385	6	male	1		yes		Parachlamydiaceae	
1463	7	female	1		yes		Parachlamydiaceae	*M. pneumoniae* IgM+
1510	7	male	1		yes		Parachlamydiaceae	
V15	13	female	2	1	NA		Parachlamydiaceae	
V45	14	female	2	1	yes	fever, cough, rhinitis	Parachlamydiaceae	
V8	13	male	2	1	NA		Parachlamydiaceae	*M. pneumoniae* PCR+
V48	14	female	2	1	yes	fever, cough, rhinitis	Parachlamydiaceae	
V57	14	male	2	1	NA		Parachlamydiaceae	
V62	9	male	2	2	yes	cough, rhinitis	Parachlamydiaceae	
V71	9	male	2	2	no		Parachlamydiaceae	
V104	12	male	2	2	yes	cough, rhinitis	Parachlamydiaceae	
V111	17	female	2	3	yes	fever, cough, rhinitis	Parachlamydiaceae	
V114	14	female	2	1	NA		Parachlamydiaceae	
K4	9	female	2	4	yes	fever, cough	Parachlamydiaceae	
V230	17	female	2	3	NA		Parachlamydiaceae	
V74	9	male	2	2	yes	fever	Criblamydiaceae	
K13	10	male	2	4	yes	fever, cough, rhinitis	Criblamydiaceae	
V211	11	female	2	2	yes	rhinitis	Simkaniaceae	
V42	14	female	2	1	yes	cough, rhinitis	Unclassified	
V49	14	male	2	1	yes	fever	Unclassified	

* 1 = Sought healthcare, 2 = Screening in schools. ** 1 = Virolahti Middle School, 2 = Virojoki School, 3 = Virolahti High Shool, 4 = Klamila School.
